# Distinctive Pattern of Serum Elements During the Progression of Alzheimer’s Disease

**DOI:** 10.1038/srep22769

**Published:** 2016-03-09

**Authors:** Giuseppe Paglia, Oto Miedico, Adriana Cristofano, Michela Vitale, Antonella Angiolillo, Antonio Eugenio Chiaravalle, Gaetano Corso, Alfonso Di Costanzo

**Affiliations:** 1 Center for Biomedicine, European Academy of Bolzano/Bozen, Via Galvani 31, 39100 Bolzano, Italy; 2 Istituto Zooprofilattico Sperimentale di Puglia e Basilicata, via Manfredonia 20, 71121, Foggia, Italy; 3 Centre for Research and Training in Medicine for Aging, Department of Medicine and Health Sciences “V Tiberio”, University of Molise, Via De Sanctis 1, 86100, Campobasso, Italy; 4 Department of Clinical and Experimental Medicine, University of Foggia, Viale L. Pinto 1, 71122, Foggia, Italy

## Abstract

Element profiling is an interesting approach for understanding neurodegenerative processes, considering that compelling evidences show that element toxicity might play a crucial role in the onset and progression of Alzheimer’s disease (AD). Aim of this study was to profile 22 serum elements in subjects with or at risk of AD. Thirtyfour patients with probable AD, 20 with mild cognitive impairment (MCI), 24 with subjective memory complaint (SMC) and 40 healthy subjects (HS) were included in the study. Manganese, iron, copper, zinc, selenium, thallium, antimony, mercury, vanadium and molybdenum changed significantly among the 4 groups. Several essential elements, such as manganese, selenium, zinc and iron tended to increase in SMC and then progressively to decrease in MCI and AD. Toxic elements show a variable behavior, since some elements tended to increase, while others tended to decrease in AD. A multivariate model, built using a panel of six essential elements (manganese, iron, copper, zinc, selenium and calcium) and their ratios, discriminated AD patients from HS with over 90% accuracy. These findings suggest that essential and toxic elements contribute to generate a distinctive signature during the progression of AD, and their monitoring in elderly might help to detect preclinical stages of AD.

Among the primary challenges of modern medicine there is cure and prevention of Alzheimer’s disease (AD), the most frequent and fearsome form of dementia. In 2015, AD and other causes of dementia affected 46.8 million people worldwide with an increasing global prevalence estimated by the year 2050 to reach 131.5 million people^1^.

Now, it is understood that preclinical stage of AD can begin more than a decade before symptoms are evidents, and therefore detection of AD as soon as possible appears to be a critical factor to fight the disease^2^ . Currently, two clinical conditions, known as mild cognitive impairment (MCI) and subjective memory complaint (SMC), have been identified as the earliest stages of disease. People affected by MCI experience memory loss and/or other cognitive impairments greater than that expected based on age and level of education, but not enough to allow a diagnosis of dementia^3^,^4^ . On the other hand, SMC subjects report memory problems that are not always perceived by others and that are not assessable with neuropsychological tests^5^,^6^. In elderly, both MCI and SMC are associated with an increased risk of dementia and could benefit from early treatment^3^,^4^,^5^,^6^. Nowadays, no marker can be used as AD indicators in these early stages and the diagnosis of AD is still based on clinical ground^7^. Biomarkers capable of identifying preclinical state of AD are in the hope of outlining a therapeutic window in which the neural substrate remains responsive to treatment. Furthermore, biomarkers capable of defining the at risk state, may drive to novel therapeutic strategies to finally achieve the disease-modifying status of AD^8^.

Recently, Cavaleri proposed a paradigm shift in AD^9^, redefining the pathomechanism in which it is included the contribution of transition metals. The literature indicates that uncontrolled oxidation and downstream inflammation play critical roles in the early stages of the disease by setting in motion its development, and shows that heavy metals can play a significant role in amyloid deposition^10^,^11^. The proposed new paradigm highlights the protective role of beta amyloid cleaving enzyme-1 (BACE1)/beta-amyloid protein system against toxic elements and oxidation. Beta-amyloid might serve as a chelating protein designed to protect the neuron from toxic metals that can exacerbate oxidative load *via* the Fenton reaction. In later stages, the uncontrolled metals and reactive oxygen species (ROS) overcame the countermeasure of BACE1/β-amyloid protein, leading to Tau hyperphosphorylation with sudden cytoskeletal dysfunction and irreversible neuron loss^9^. Gonzalez-Dominguez *et al*.^12^, profiled 13 elements in the serum of patients with MCI and AD, reporting altered concentration of some elements that could be related to the development and the progression of AD. Considering that impaired element homeostasis seems closely related to AD and to its preclinical stages, the characterization of metal profiles in patients with or at risk of dementia appears to be of special interest for understanding the pathogenesis of the disease and for the identification of biomarkers.

In the present study, we analyzed serum elements along the continuum from healthy subjects (HS), through patients suffering of SMC and/or MCI, up to those with AD. We profiled 22 elements by inductively coupled plasma mass spectrometry (ICP-MS) with a double purpose: to understand if the homeostasis of essential and toxic elements is implicated in the onset and progression of AD, and to evaluate the analyzed elements as possible diagnostic biomarkers for the disease.

## Results

Clinical and demographic characteristics of the recruited population are reported in Table 1. The serum concentration of the 22 elements profiled are reported in Tables 2 and 3. For 16 elements, over 94% of the measurements were greater than limit of quantification (LOQ). For vanadium (V), tin (Sn) and chromium (Cr) we obtained 65%, 68%, and 53% of data higher than LOQ. For aluminum (Al), beryllium (Be) and cadmium (Cd) we recorded more than 70% of data lower than LOQ; therefore these elements were not further considered in the results and discussion sections.

We first performed analysis of variance, including age, gender, level of education, BMI, presence of heart failure or chronic kidney disease and use of diuretics or supplements containing metals as covariates, which resulted in a statistically significant difference (F = 3.080; df = 39, 288; p < 0.001; Pillai’s Trace = 0.833; partial η^2^ = 0.294). Ten elements, manganese (Mn), V, selenium (Se), mercury (Hg), zinc (Zn), molybdenum (Mo), thallium (Tl), iron (Fe), copper (Cu), and antimony (Sb), changed significantly among the 4 groups (Tables 2 and 3). Pairwise multiple comparisons showed that five elements (Mn, V, Hg, Mo and Sb) significantly changed in AD, three (Mn, Se and V) in MCI and two (Cu and Sb) in SMC, compared to HS (Tables 2 and 3). Other significant pairwise comparisons are reported in Tables 2 and 3.

### Elements Signature during the progression of AD

**Figure 1:**
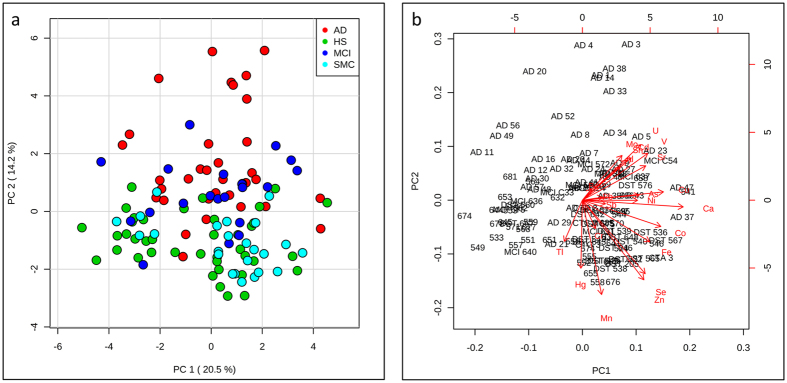
Principal Component Analysis (PCA) (a) PCA separates AD samples from the other groups. (**b**) Loading biplot shows how essential elements and toxic elements contribute in a different way to AD samples clustering.

Principal component analysis (PCA) showed that first and second principal components (PC1 and PC2) tend to separate the AD class from HS, while both MCI and SMC classes tend to cluster in an intermediate zone (Fig. 1a). Both essential, such as Se, Zn, and Mn, and toxic elements, such as V, strontium (Sr), Sn and uranium (U), strongly influenced the clustering of AD samples. However essential and toxic elements provided a different contribution to the separation, particularly in the PC2 (Fig. 1b). This is confirmed by the PC2 loadings reported in supplementary Table S1, where it is shown that many of the essential and toxic elements had a different trend: negative values for essential elements and positive values for toxic elements.

**Figure 2:**
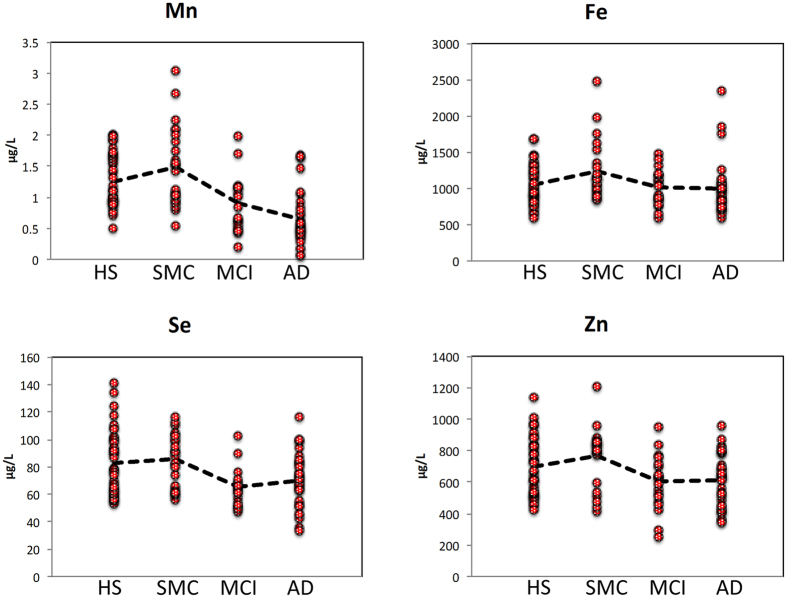
Profiles of selected essential elements. Essential elements show a similar profile with highest values in SMC samples and lowest values in AD samples. Dot line represents the average value.

**Figure 3:**
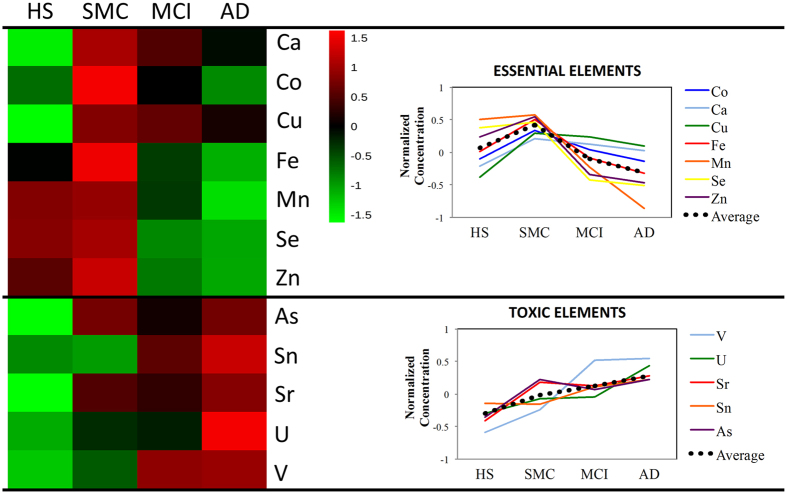
Heatmap. Essential elements showed a characteristic pattern, which was different from the one of toxic elements. The average values were used after data normalization as described in methods.

**Figure 4:**
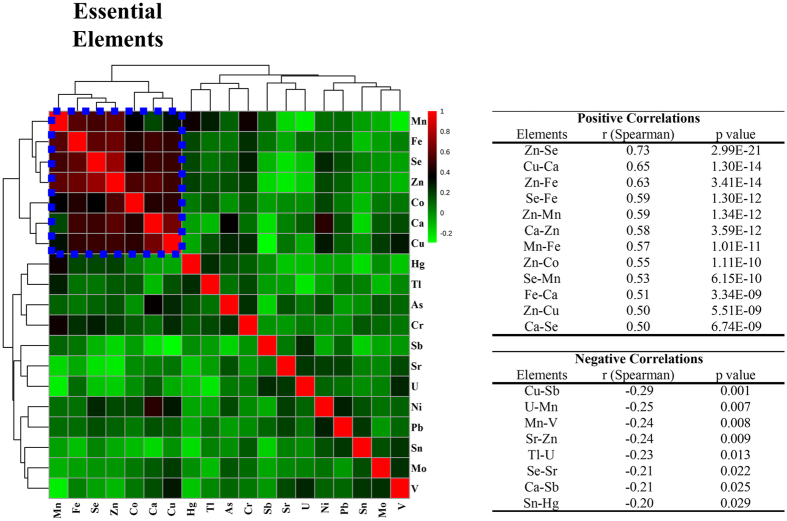
Correlation analysis. Essential elements strong correlate between each other. In the table are reported positive and negative significant correlations.

Essential elements (with the exception of Mo) exhibited a similar profile describing a distinctive element signature during the progression of cognitive disturbances from HS to AD patients (Figs 2 and 3). In particular, the serum concentration of Se, Zn, Mn and Fe had a characteristic profile in which these elements increased in the SMC patients and then progressively decreased in MCI and in AD patients (Fig. 2). This finding was further confirmed by correlation analysis (Fig. 4) showing that Se, Mn, Zn, and Fe well correlated with each other as well as with calcium (Ca) and Cu (r > 0.5).

In contrast, many toxic elements had a completely different profile, such as V, U, Sr, Sn, and As, resulting in a progressive accumulation along the different stages of the disease (Fig. 3). However, correlations among toxic elements were not as evident as for essential elements (Fig. 4).

### Biomarkers Validation

**Figure 5:**
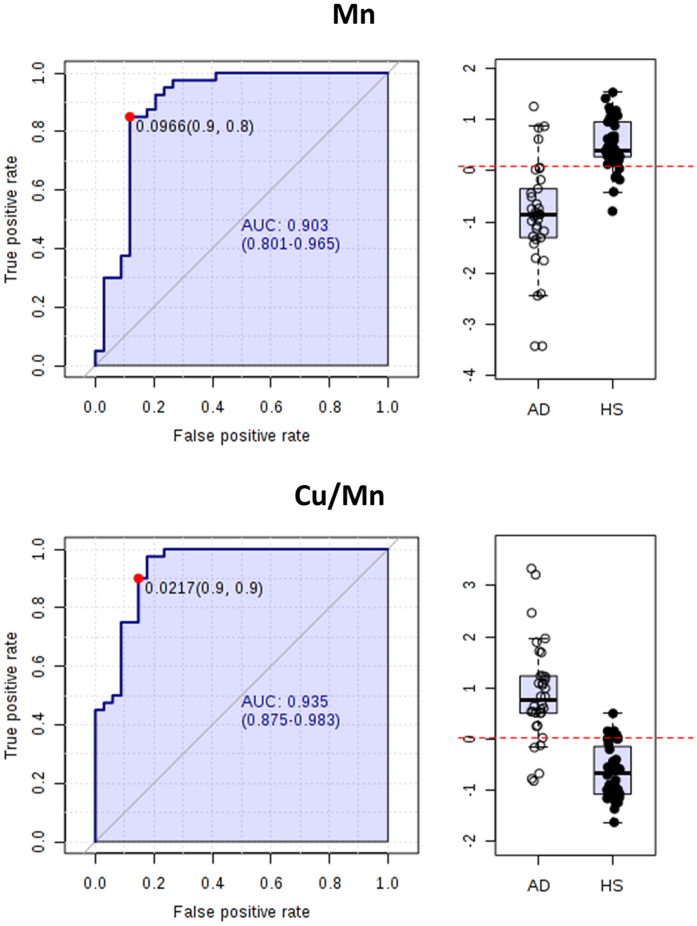
Univariate ROC curves analysis individuated Cu and the ratio Cu/Mn as two potential biomarkers for discriminating between AD and HS samples. AUC = Area under the curves. The confidential interval is also provided. Plot bars were obtained using normalized concentration as described in methods.

The results of univariate receiver operating characteristic (ROC) curve analysis are reported in Table 4. We selected the biomarkers with the area under the curve (AUC) higher then 0.7 and statistical power higher than 70%. Al, Be and Cd were excluded because 70% of measurements were lower than LOQ. As general trend, AUC and p values resulted higher for ratios. In the discrimination between AD and HS, the two elements having the best diagnostic power were Mn (AUC = 0.89) and V (AUC = 0.83). The essential elements Zn and Se also showed a good discriminating power (Table 4). Considering the specific trend of biomarkers such as Mn (AUC = 0.89) and the ratio Cu/Mn (AUC = 0.93), we individuated these two parameters as good potential biomarkers for discriminating HS from AD patients (Fig. 5).

As regard the discrimination among the other groups, Cu (AUC = 0.70) is a potential candidate to discriminate SMC from HS, and three essential elements (Mn, AUC = 0.80; Se, AUC = 0.79; Zn, AUC = 0.74) seem to be able to distinguish MCI from SMC samples (Table 4 and Supplementary Fig. S1).

**Figure 6:**
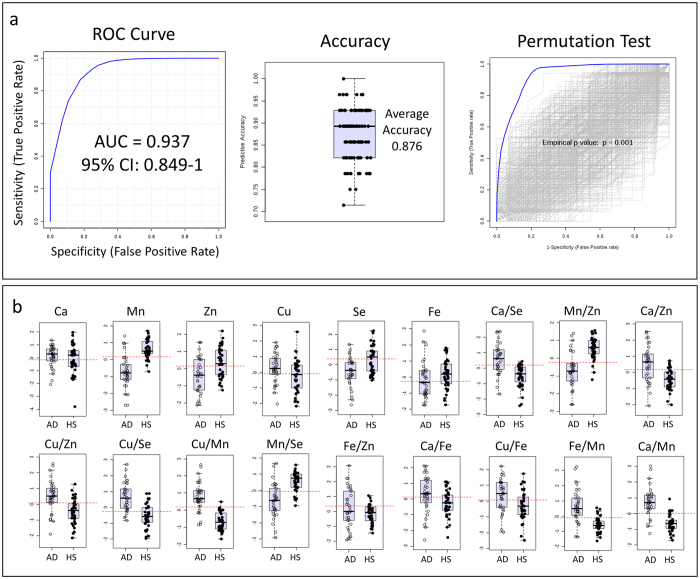
Multivariate ROC curves analysis was performed by generating a model with 6 essential elements (Cu, Ca, Mn, Zn, Fe and Se) and their ratios. (**a**) Random Forest algorithm was used during this analysis. AUC = Area under the curve. CI = Confidential Intervals. The model was then validated by cross validation and permutation test. (**b**) Selected features included in the model (Supplementary Table S2).

Multivariate ROC curves analysis was performed to better differentiate AD from HS samples. The six essential elements Se, Mn, Zn, Ca, Fe and Cu, together with their respective ratios, (overall 21 features) were included in the analysis (Fig. 6 and Supplementary Table S2). The selected essential elements had good discriminating power (Tables 2, 3, 4 and Fig. 5) as well as high correlation coefficients with each other (Fig. 4). The resulting classification/regression model had a strong diagnostic power with an AUC of 0.937 (Fig. 6a). The cross-validation by Random Forests algorithm showed an average accuracy of 0.876 (Fig. 6a). In the permutation tests, none of the results was better than the original one, obtaining a p < 0.001 (Fig. 6a). In the Fig. 6b are reported the most significant biomarkers and their ratios which showed an AUC higher than 0.64.

## Discussion

Several studies have reported the levels of elements in biological fluids (serum, plasma, blood and/or cerebrospinal fluid) and in various brain regions of patients suffering from defects of cognitive functions. Unfortunately, most of these studies reported the analysis of a single element or few of them, measured by diverse methods and technologies^13^,^14^,^15^,^16^,^17^. Therefore, it is difficult to compare results among different studies, also considering that often results do not agree with each other. On the other hand, systematic studies analyzing a wider panel of elements (19–22 elements) in serum or plasma with standardized methodologies are limited^12^,^18^,^19^,^20^. Most of these studies examined patients with AD or MCI, but to best of our knowledge, none of them has investigated SMC subjects. Therefore, a comparative study among patients with SMC, MCI and AD is of great importance. Such a study can provide information on the neurodegenerative progression, on the pathogenesis of disease and also can drive to the identification of potential biomarkers for early diagnosis.

In the present work, we analyzed 22 serum elements by ICP-MS in HS and patients affected by three different cognitive defects (SMC, MCI and AD). Statistical analysis revealed that the four investigated groups differed significantly for 10 elements (Tables 2 and 3). In particular, pairwise comparisons showed that 5 elements (Mn, V, Hg, Mo and Sb) changed significantly in AD, 3 in MCI (Mn, V and Se) and 2 in SMC (Sb and Cu), compared to HS. Furthermore, 7 elements (Mn, V, Se, Hg, Zn, Tl and Fe) changed significantly between AD and SMC, 2 elements (Hg, and Mn) between AD and MCI, and 4 between MCI and SMC (Mn, V, Se and Zn). Interestingly to note that both essential and toxic elements had a different trend profile when passing from HS through SMC and MCI, up to AD (Figs 2 and 3, and Tables 2 and 3). In particular, Mn, Zn, Se and Fe increased of 20%, 9%, 3% and 14%, from HS to SMC. Then they progressively decreased in MCI by −27%, −13%,−21% and −3%, and in AD by −52%, −13%, −15% and −10%, respectively (Fig. 2).

The trend toward low serum levels of these elements in AD is in agreement with several studies^12^,^18^,^21^,^22^,^23^,^24^, although higher or unchanged levels of Mn, Fe, Se and Zn were also detected^18^,^20^,^21^,^25^. The trend reporting lower amount of Mn, Zn and Fe in total serum of MCI samples compared with HS is in line with other studies^12^,^26^,^27^. Gonzalez-Dominguez *et al*. measured elements in different serum fractions, low molecular mass (LMM) and high molecular mass (HMM), reporting a different trend for Fe and Zn. Indeed, in HMM serum fractions, Fe and Zn decreased in AD when compared to HS, while the same elements increased in LMM serum fractions of AD patients^12^. At contrary, LMM serum fractions of Fe resulted higher than normal in MCI population^12^.

Cu concentration increased in SMC and slightly decreased in MCI and AD, although remaining higher than in HS (Fig. 3 and Table 2). The trend towards high levels of Cu in AD is in line with some reports^17^,^28^. According to our findings, several studies did not report significant changes for Ca^20^,^21^,^29^,^30^ and Co^12^ in the AD. However, increased levels of Ca^18^,^19^ and decreased levels of cobalt (Co)^18^,^19^,^20^ were also detected. The correlation analysis showed that Fe, Se, Mn and Zn well correlated with each other as well as with Ca and Cu (r > 0.5; Fig. 5), suggesting that these elements are interrelated, participating in common metabolic processes and contributing to ongoing pathological features of AD.

Using multivariate analysis (PCA), we separated AD samples from HS, SMC and MCI (Fig. 1a), and we found that many essential elements, such as Se, Zn, Fe, and Mn provided a different contribution to the separation when compared to many toxic elements, such as V and U (Figs 1b and 3 and Supplementary Table S1).

The only essential element showing a different behavior was Mo; in fact, its level increased progressively, passing from HS through SMC, MCI, up to AD, and the difference between HS and AD was statistically significant (Table 2). Its levels were previously found increased in AD^19^ but this finding was not further confirmed^12^,^18^,^27^.

As regard to toxic elements, 5 of them (V, Sr, Sn, As, and U) showed an upward trend from HS to AD (Fig. 3). This finding is in line with previous studies reporting increased levels of V^12^ and Sn^18^,^19^, in serum or plasma of AD patients (Table 3). In contrast with previous studies reporting similar^31^ or higher^18^,^20^,^21^ serum levels of Hg in AD vs. HS, we found that Hg profile is comparable to some essential elements, (i.e. increasing in SMC and progressively decreasing in MCI and AD) (Table 2). However, the relationship between Hg and AD is not clear, and a recent review on this topic concluded that the measurements of Hg levels in blood, urine, hair, nails and cerebrospinal fluid are currently inconsistent^32^. Sb showed a progressive decrease from HS, through SMC and MCI, up to AD (Fig. 3) in disagreement with previous studies that did not report differences between AD and HS^18^,^19^,^20^. According to others, lead (Pb)^12^,^18^,^19^,^20^,^31^ and Tl^18^,^19^ did not show any change. Correlation analysis showed that toxic elements were weakly correlated between each other, suggesting a variable and inconstant role in the pathogenesis of AD (Fig. 4). There are several potential reasons for explaining this discrepancy in the results reported in the literature. In fact, this disagreement might be due to the complex multifactorial and multi-genetic nature of AD, to differences in number and clinical characteristics of the population examined and to the use of different methodological approaches.

We also evaluated the diagnostic power of elements, selecting biomarkers with an AUC higher than 0.7 and statistical power higher than 70%. In addition, we calculated the ratios between the most important and best correlated elements. Mn, V and the ratio Cu/Mn showed the best diagnostic power in the discrimination between AD and HS with AUCs of 0.89, 0.83 and 0.93, respectively (Table 4). Furthermore, ROC analysis showed the possibility to discriminate HS from SMC samples by the serum levels of Cu (AUC = 0.70) and Sb/V ratio (AUC = 0.71), as well as MCI from SMC by Mn (AUC = 0.80), Se (AUC = 0.79) and Zn (AUC = 0.74) and V (AUC = 0.73). In Table 4 are also reported all the best AUC values obtained from the comparisons of all studied groups.

For a better distinction of AD from HS samples, we performed a ROC analysis based on multivariate algorithm (Random Forest) to generate cross-validated ROC curves for more realistic prediction. The analysis was performed on six essential elements, such as Se, Mn, Zn, Ca, Fe and Cu, showing high discriminating potentials and high correlation coefficients, and their respective ratios. The built classification model, including 21 features, showed a high diagnostic and validated power with an AUC of 0.937 and an average accuracy of 0.876 (Fig. 6a).

The findings herein described support the hypothesis that there has to be a crucial role of elements in the development of AD, a role that involves both toxic and essential metals^13^,^33^, even if the most interesting results were obtained for essential elements. However, considering that the degree of exposure to environmental and nutritional factors for both patients and controls is unknown, we believe that it is not possible to make an accurate discussion on the liaison between elements and AD. Further studies should take into account this aspect before analyzing the relationships among metabolites, elements and other environmental or nutritional factors in the disease.

Anyway, toxic elements, such as Pb, Hg, Al, Cd and As, are implicated in AD pathogenesis due to their ability to promote the beta-amyloid production and the phosphorylation of Tau protein, and therefore the formation of amyloid plaques and neurofibrillary tangles (NFTs) characteristic of disease. Another mechanism implicated is the metal-induced oxidative stress^16^. Essential elements are also implicated in the pathogenesis of AD, since they can produce similar effects, such as amyloid plaques and neurofibrillary tangles formation and oxidative stress^34^,^35^,^36^,^37^,^38^. However, it is also well studied the protective role of some essential elements in AD, such as the anti-oxidant function of Se, as well as the neuro-protective role of Zn against beta-amyloid cytotoxicity^39^,^40^.

In conclusion, the results of the present study suggest that essential elements are strictly interrelated and contribute to a distinctive signature during the progression of AD. The most significant changes were observed in Mn, Fe, Se and Zn homeostasis, which tend to increase in SMC and then progressively decrease in MCI and AD. Toxic elements show a variable behavior, since some elements tend to increase, while others tend to decrease in AD. Furthermore, a serum panel of six essential elements and their ratios robustly differentiates AD patients from HS (with accuracy above 90%). However, this biomarker panel requires further validation on a larger number of samples in a longitudinal study before further development for clinical use.

## Methods

### Participants

We collected a total of 118 serum samples including 40 HS (gender: 15 males and 25 females; age: mean ± SD, 65.53 ± 6.37 years), 24 patients affected by SMC (10 males and 14 females; 68.04 ± 8.05 years), 20 patients affected by MCI (4 males and 16 females; 68.30 ± 7.75 years) and 34 patients affected by AD (9 males and 25 females; 72.44 ± 7.48 years). Subjects were consecutively enrolled from the Centre for Research and Training in Medicine for Aging (CeRMA), University of Molise (Italy). Patients with probable AD were diagnosed according to National Institute of Neurological and Communicative Diseases and Stroke/Alzheimer’s Disease and Related Disorders Association (NINCDS-ADRDA) criteria^41^, and presented Mini Mental State Examination (MMSE)^42^ score <24, and Clinical Dementia Rating (CDR)^43^ score > 0.5. Subjects with amnestic MCI met the Petersen’s diagnostic criteria^3^ , had MMSE > 24 and CDR = 0.5, and showed memory impairment as assessed via age-sex-education-adjusted scores on at least one of the following tests: Rey’s word list immediate and delayed recall^44^ and Prose memory, immediate and delayed^45^. Participants with SMC stated that their memory function has deteriorated compared to earlier stages in life, reported that the time of onset was in adulthood, had a score of 25 or more on the Memory Complaint Questionnaire (MAC-Q)^46^ and showed normal objective memory performance on Rey’s and Prose memory tests^5^. To summarize, MCI subjects showed both subjective and objective memory impairment, SMC participants presented only memory complaints with a normal score on the memory tests and HS showed neither subjective nor objective memory impairment. Depression at screening was assessed with the Geriatric Depression Scale (GDS)^47^, and participants with a GDS score of 6 or more were considered depressed and excluded from the study. Sixteen patients were on treatment with nootropic drugs and they underwent to a wash-out period of at least 14 days before assessment and blood sampling.

Experiments and methods of this study were conducted in accordance with ethical principles stated in the Declaration of Helsinki, as well as with approved national and international guidelines for human research. The Ethics Committee of University of Molise reviewed and approved this study and a written informed consent was required from participants or caregivers.

### Blood samples

Blood collection was done between 8:00 and 8:30 AM after an overnight fast. Venous blood was collected into vacutainer tubes (Becton & Dickinson) and centrifuged within four hours. All serum samples were stored at −80 °C until the shipment to the analytical laboratory. Serum samples were packed with dry ice and sent by courier to the laboratory. The analytical laboratory was blinded to sample identification codes.

### Chemicals

HNO_3_ (68%, v/v), H_2_O_2_ (30% v/v) and Ultrapure Water were purchased from Romil Ltd (Cambridge, UK); Al, Sb, As, Be, Cd, Ca, Cr, Co Cu, Fe, Pb, Hg, Mn, Mo, nickel (Ni), Se, Sr, Tl, Sn, U, V and Zn standard solutions (1000 mg/l) were purchased from CPA Ltd (Stara-Zagora, Bulgaria). Anhydrous ammonia and methane (99.9995%) were purchased from AIR Liquide S.p.a. (Milan, Italy); ultrapure Argon (99.9999%) was purchased from SAPIO S.r.l. (Monza, Italy).

### Elements Determination by ICP-MS

A modified version of an optimized method^48^ was used in this study. A quick and simple treatment was applied: after shaking and whirling the entire sample, 0.50 mL of serum sample was diluted 10 times by adding 4.5 mL of an aqueous solution of HNO_3_ 1% (v/v). Samples were then passed to the vortex for 1 min and analyzed by ICP-MS. An inductively coupled plasma mass spectrometer (Elan DRC II PerkinElmer, Waltham, Massachusetts, USA) equipped with a concentric nebulizer (Meinhard Associates, Golden, USA), a cyclonic spray chamber (Glass Expansion, Inc., West Merbourne, Australia) and a quartz torch with a quartz injector tube (2 mm i.d.) was used. The operational parameters were set at 1200 Watt for radio frequency, 15 l/min for plasma gas (Ar), 0.97 L/min for nebulizer gas (Ar), 60 sec of sample flush, 32.0 rpm for sample flush speeding, 20 sec of read delay, 20 rpm for read delay and analysis speeding, 45 sec for wash, 32 rpm for wash speeding, 50 ms of dwell time, 20 sweeps/reading, Rhodium and Bismuth as internal standards, added to standard and sample solution by on-line mixing.

The following elements/isotopes were detected: ^8^Be, ^27^Al, ^44^Ca, ^51^V, ^52^Cr, ^55^Mn, ^56^Fe, ^59^Co, ^60^Ni, ^63^Cu, ^66^Zn, ^75^As, ^78^Se, ^88^Sr, ^98^Mo, ^111^Cd, ^118^Sn, ^120^Sb, ^202^Hg, ^205^Tl and ^238^U. In order to eliminate the intrinsic variability of lead isotope distribution and to improve the signal sensitivity, the sum of ^206^Pb, ^207^Pb and ^208^Pb were counted. In order to minimize isobaric interference, the Dynamic Reaction Cell (DRC) system was used employing ammonia gas (100%, high purity) at 0.5 mL/min for the determination of Al, As, Co, Cr, Cu, Fe, Mn, Ni, V and Zn, and methane gas at 0.5 mL/min for the determination of Se. The instrumental calibration was performed by standard addition into the diluted and acidified solution: for each element 5 addition levels, including the not added level, were used. The addition levels were stated on the basis of literature data^12^,^48^,^49^. A mixed serum sample, after 10 times dilution, was used to obtain the spiked standards. The addition levels for each element were: U, Hg, Tl, Cd, V, Be (5 – 25 – 125–500 ng/L); Pb, Al, As, Sb, Ni, Co, Mo, Sn, Mn, Cr (0.02-0.1-0.5-2 μg/L); Se, Sr (1-5-25-100 μg/L); Cu, Zn, Fe (10-50-250-1000 μg/L); Ca (500-2500-12500-50000 μg/L). The linearity was studied adopting 5 calibration levels (as described above). A good linearity was observed in the calibration range set for each element with determination coefficient (R^2^). The goodness-of-fit of the data to the calibration curve has been checked by Mandel test.

Limit of detection (LOD) values of the method for each element were determined by blank determination assays, as 3 times standard deviation of 20 replicates and were reported in ng/l considering the diluted solution. Analogously, limit of quantification (LOQ) values were calculated as 10 times the standard deviation of 20 blank replicates and they were reported in μg/L referring to serum sample, considering the dilution step occurred in the preparation (0.50 mL of serum sample diluted 10 times).

### Statistical analysis

Data was analyzed as first step, using the SPSS (v. 17.0) statistical software package (SPSS Inc., Chicago, Ill.). Variables were examined for outliers and extreme values by means of box and normal quantile-quantile plots, and Shapiro-Wilk’s and Kolmogorov-Smirnov’s tests. When normal distribution could not be accepted, variable transformations (square, square root, logarithmic, reciprocal of square root or reciprocal transformations) were reviewed. The reciprocal of square root of As, Fe, Pb, Sr, Tl levels, the square root of Hg, Mn, Ni, Se, Zn concentrations and the logarithm of Mo levels helped to improve the distribution shape. However, normal distribution could not be reached for Ca, Cr, Sb, U, V variables.

One-way multi- and univariate analysis of variance (ANOVA), with age, gender, education level, BMI, presence of heart failure or chronic kidney disease, and use of diuretics or supplements containing metals (Ca, Fe, Se and Zn) as covariates, for normally distributed variables, and the Kruskal-Wallis H test for not normally distributed variables were conducted to determine group differences (HS vs. SMC vs. MCI vs. AD). Moreover, for normal variables, the assumption of equality of variance was assessed by means of Levene’s test. Finally, post hoc pairwise multiple comparisons, using Bonferroni’s correction for normal and Dunn’s procedure for not normal variables were performed in order to detect significant differences between two specific groups.

Correlation analysis was performed by using MetaboAnalyst^50^. Spearman rank order correlation was used to measure the correlation. Power analysis was performed post hoc by calculating the statistical power for each element using one-tail test and 95% of confidence interval (Alpha = 5%).

Principal component analysis (PCA) was performed using MetaboAnalyst^50^ in order to individuate any variation in the obtained dataset. Before PCA, data were batch normalized as previously described^51^ dividing each variable of each batch by the square root of the sum of the squares of all original values of that batch. Finally the dataset was log transformed and scaled by using unit variance scaling method (mean-centered and divided by the standard deviation of each variable).

The heatmap was obtained by using the average values of selected essential and toxic elements. Before calculating the average, the batch of data was normalized as described above. Univariate ROC curves analysis was performed to evaluate the diagnostic power of all elements and ratios by using ROCCET^52^. Before univariate ROC curves analysis data was batch normalized as described previously^51^, by dividing each variable of each batch by the square root of the sum of the squares of all original values of that batch. Finally the dataset was log transformed and scaled by using unit variance scaling method (mean-centered and divided by the standard deviation of each variable).

Multivariate ROC curves analysis was performed on 6 selected essential elements (Mn, Zn, Se, Cu, Fe and Ca) and their ratios by using ROCCET^52^. The performance of the generated model was first cross-validated by using Random Forests. In brief, the model was validated through repeated random sub-sampling cross validation where in each cross validation, two thirds of the samples are used to evaluate the importance of each feature based on decreases in accuracy. The generated model was then further validated by 1000 permutation tests using the area under the curve as performing measure. Before multivariate ROC curves analysis, data was batch normalized as previously described^51^, by dividing each variable of each batch by the square root of the sum of the squares of all original values of that batch. Finally the dataset was log transformed and scaled by using unit variance scaling method (mean-centered and divided by the standard deviation of each variable).

## Additional Information

**How to cite this article**: Paglia, G. *et al*. Distinctive Pattern of Serum Elements During the Progression of Alzheimer’s Disease. *Sci. Rep.* **6**, 22769; doi: 10.1038/srep22769 (2016).

## Supplementary Material

Supplementary Information

## Figures and Tables

**Table 1:** Demographic and clinical characteristics of the study groups.

		Group			
		HS	SMC	MCI	AD
		(n = 40)	(n = 24)	(n = 20)	(n = 34)
Age (mean ± SD, range, y)		65.53 ± 6.37	68.04 ± 8.05	68.30 ± 7.75	72.44 ± 7.48
		(57 to 87)	(54 to 87)	(54 to 84)	(54 to 84)
Gender (n, %)	Male	15; 37.5%	10; 41.7%	4; 20%	9; 26.5%
	Female	25; 62.5%	14; 58.3%	16; 80%	25; 73.5%
Education level (mean ± SD, range,y)		12.75 ± 3.16	12.00 ± 3.54	9.75 ± 3.86	8.65 ± 4.47
		(5 to 18)	(5 to 18)	(5 to 17)	(3 to 18)
BMI (mean ± SD, range, Kg/m^2^)		29.09 ± 4.28	26.98 ± 3.47	27.97 ± 3.53	25.76 ± 5.51
		(22.04 to 39.91)	(21.78 to 34.10)	(21.36 to 34.34)	(17.87 to 37.55)
MMSE (mean ± SD, range)		29.57 ± 0.75	29.6 ± 0.94	26.28 ± 3.99	12.31 ± 8.15
		(27 to 30)	(27 to 30)	(24 to 30)	(0 to 23)
Medical History (n, %)					
Smoke^a^		10; 25.0%	4; 16.7%	4; 20.0%	10; 29.4%
Dyslipidemia		12; 30.0%	6; 25.0%	5; 25.0%	12; 35.3%
Diabetes		4; 10.0%	3; 12.5%	3; 15.0%	7; 20.6%
Hypertension		17; 47.5%	9; 37.5%	10; 50%	17; 50%
Arrhythmia		3; 7.5%	2; 8.3%	2; 10.0%	2; 5.9%
Myocardial infarction		1; 2.5%	3; 12.5%	1; 5%	4; 11.8%
Heart failure^b^		1; 2.5%	–	–	2; 5.9%
TIA/Stroke		–	2; 8.3%	1; 5.0%	2; 5.9%
Chronic kidney disease^c^		–	–	–	1; 2.9%
Prior Tumors		5; 12.5%	4; 16.7%	1; 5.0%	3; 8.8%
Drugs (n, %)					
Antihypertensive		17; 40.0%	9; 37.5%	10; 50.0%	17; 50.0%
Diuretic		7; 17.5%	5; 20.8%	4; 20.0%	6; 17.6%
Lipid-lowering		7; 17.5%	6; 25.0%	3; 15.0%	5; 14.7%
Hypoglycemic		4; 10.0%	3; 12.5%	3; 15.0%	5; 14.7%
Antiplatelet		5; 12.5%	5; 20.8%	2; 10.0%	8; 23.5%
Supplements containing metals		6; 15.0%	5; 20.8%	3; 15.0%	5; 14.7%

^a^current or former smoker.

^b^subjects in NYHA (New York Heart Association) class I-II.

^c^subjects with glomerular filtration rate 60 mL/min/1.73m2>(GFR)>30 mL/min/1.73m2; –, none; AD, Alzheimer disease; MCI, mild cognitive impairment; SMC, subjective memory complaint; HS, healthy subjects; BMI, Body mass index; MMSE, Mini Mental State Examination; TIA, transient ischemic attack.

**Table 2:** Element concentrations (μg/L) in serum of patients and healthy subjects.

Element	HS	SMC	MCI	AD	ANCOVA^a^		Pairwise comparisons^b^*(p value)*					
	(n = 40)	(n = 24)	(n = 20)	(n = 34)	*F(3,106)*	*p value*	ADvsHS	ADvsSMC	ADvsMCI	HSvsSMC	HSvsMCI	MCIvsSMC
As	3.13 ± 0.75(2.12 to 5.35)	3.32 ± 0.60(2.05 to 4.56)	3.39 ± 0.69(2.46 to 5.06)	3.55 ± 1.08(2.20 to 5.99)	1.204	0.312	N/A	N/A	N/A	N/A	N/A	N/A
Co	0.39 ± 0.07(0.23 to 0.56)	0.41 ± 0.06(0.29 to 0.51)	0.39 ± 0.06(0.27 to 0.49)	0.39 ± 0.09(0.25 to 0.68)	0.614	0.607	N/A	N/A	N/A	N/A	N/A	N/A
Cu	703.88 ± 244.03(358 to 1460)	858.96 ± 224.19(335 to 1310)	826.59 ± 235.46(407 to 1293)	815.75 ± 206.00(370 to 1210)	3.013	0.033	n.s.	n.s.	n.s.	0.049	n.s.	n.s.
Fe	1045.07 ± 271.05(594 to 1690)	1192.72 ± 284.39(852 to 1780)	1019.69 ± 247.51(584 to 1470)	938.54 ± 209.79(590 to 1440)	2.891	0.039	n.s.	0.003	n.s.	n.s.	n.s.	n.s.
Hg	0.62 ± 0.28(0.18 to 1.60)	0.69 ± 0.33(0.21 to 1.42)	0.67 ± 0.43(0.19 to 1.37)	0.32 ± 0.27(0.06 to 1.09)	8.732	<0.001	<0.001	<0.001	<0.001	n.s.	n.s.	n.s.
Mn	1.24 ± 0.42(0.49 to 2.00)	1.49 ± 0.64(0.53 to 3.04)	0.91 ± 0.48(0.21 to 1.98)	0.59 ± 0.32(0.06 to 1.18)	14.783	<0.001	<0.001	<0.001	0.030	n.s.	0.024	0.001
Mo	0.83 ± 0.26(0.37 to 1.34)	0.99 ± 0.24(0.52 to 1.40)	1.09 ± 0.36(0.040 to 1.75)	1.20 ± 0.52(0.59 to 2.35)	4.199	0.008	0.001	n.s.	n.s.	n.s.	n.s.	n.s.
Ni	1.08 ± 0.38(0.38 to 2.38)	0.99 ± 0.40(0.38 to 1.96)	0.86 ± 0.27(0.38 to 1.48)	1.10 ± 0.29(0.38 to 1.74)	2.631	0.054	n.s.	n.s.	n.s.	n.s.	n.s.	n.s.
Pb	0.16 ± 0.17(0.03 to 1.05)	0.12 ± 0.13(0.04 to 0.72)	0.11 ± 0.06(0.05 to 0.26)	0.13 ± 0.09(0.03 to 0.48)	1.026	0.384	n.s.	n.s.	n.s.	n.s.	n.s.	n.s.
Se	82.62 ± 23.40(53 to 141)	85.32 ± 18.75(56 to 116)	65.41 ± 14.95(47 to 102)	70.36 ± 19.28(33 to 116)	3.199	0.026	n.s.	0.028	n.s.	n.s.	0.015	0.007
Sr	35.21 ± 9.84(19 to 67)	38.53 ± 13.10(21 to 72)	39.77 ± 12.17(27 to 81)	42.84 ± 17.01(22 to 93)	0.380	0.768	N/A	N/A	N/A	N/A	N/A	N/A
Tl	2.62 ± 1.96*(0.6 to 11.2)*	6.95 ± 13.09*(1.0 to 49.7)*	3.3 ± 3.26*(0.4 to 12.6)*	2 ± 1.96*(0.3 to 9.6)*	2.841	0.041	n.s.	0.003	n.s.	n.s.	n.s.	n.s.
Zn	697.87 ± 184.95(425 to 1140)	761.34 ± 152.83(471 to 956)	604.70 ± 176.81(249 to 947)	609.40 ± 164.31(348 to 953)	3.420	0.020	n.s.	0.008	n.s.	n.s.	n.s.	0.018

Results are reported as mean + standard deviation and (range).

^a^covariates (age, gender, education level, BMI, heart failure, chronic kidney disease, diuretics and supplements containing metals);

^b^Bonferroni post-hoc analysis; *, value are 1*10^–2^; N/A, not applicable; n.s., not significant; HS, healthy subjects; SMC, subjective memory complaint; MCI, mild cognitive impairment; AD, Alzheimer’s disease.

**Table 3:** Element levels (μg/L) in serum of patients and healthy subjects.

Element	HS	SMC	MCI	AD	Kruskal-Wallis		Pairwise comparisons^a^*(p value)*					
	(n = 40)	(n = 24)	(n = 20)	(n = 34)	*H (3)*	*p value*	ADvsHS	ADvsSMC	ADvsMCI	HSvsSMC	HSvsMCI	MCIvsSMC
Al	2.96 ± 0.76(2.71 to 5.70)	3.06 ± 0.84(2.71 to 5.60)	3.46 ± 2.04(2.71 to 10.86)	3.25 ± 1.58(2.71 to 10.94)	1.369	0.714	N/A	N/A	N/A	N/A	N/A	N/A
Be	0.02 ± 0.02(0.02 to 0.12)	0.03 ± 0.04(0.02 to 0.19)	0.02 ± 0.02(0.02 to 0.11)	0.03 ± 0.07(0.02 to 0.39)	0.180	0.981	N/A	N/A	N/A	N/A	N/A	N/A
Ca	7.2 ± 2.6*(2.67 to 10.9)*	7.9 ± 1.4*(5.16 to 9.91)*	7.7 ± 2.2*(4.12 to 10.5)*	8.1 ± 2.1*(3.99 to 10.8)*	1.163	0.762	N/A	N/A	N/A	N/A	N/A	N/A
Cd	0.02 ± 0.01(0.02 to 0.07)	0.02 ± 0.01(0.02 to 0.06)	0.03 ± 0.02(0.02 to 0.09)	0.03 ± 0.02(0.02 to0.08)	2.703	0.069	N/A	N/A	N/A	N/A	N/A	N/A
Cr	0.21 ± 0.25(0.06 to 1.19)	0.17 ± 0.13(0.06 to 0.60)	0.18 ± 0.24(0.06 to 1.14)	0.10 ± 0.06(0.06 to 0.34)	7.093	0.297	N/A	N/A	N/A	N/A	N/A	N/A
Sb	3.23 ± 0.51(2.46 to 4.77)	2.78 ± 0.48(1.55 to 3.97)	3.18 ± 1.23(1.58 to 6.34)	2.67 ± 1.29(0.04 to 5.76)	15.532	0.001	0.002	n.s.	n.s.	0.047	n.s.	n.s.
Sn	0.14 ± 0.10(0.05 to 0.39)	0.11 ± 0.09(0.05 to 0.47)	0.16 ± 0.14(0.05 to 0.49)	0.22 ± 0.21(0.05 to 0.90)	3.687	0.297	N/A	N/A	N/A	N/A	N/A	N/A
U	0.02 ± 0.01(0.01 to 0.04)	0.02 ± 0.01(0.01 to 0.08)	0.03 ± 0.02(0.01 to 0.12)	0.03 ± 0.02(0.01 to 0.09)	3.829	0.281	N/A	N/A	N/A	N/A	N/A	N/A
V	0.04 ± 0.01(0.03 to 0.09)	0.04 ± 0.02(0.03 to 0.11)	0.08 ± 0.04(0.03 to 0.20)	0.08 ± 0.04(0.03 to 0.53)	34.116	<0.001	<0,001	0.009	n.s.	n.s.	<0.001	0.016

Results are reported as mean ± standard deviation and (range). Statistical analysis has been performed by Kruskal-Wallis H test.

^a^Dunn’s post hoc analysis; N/A, not applicable;*, value are 1*10^4^; n.s., not significant; HS, healthy subjects; SMC, subjective memory complaint; MCI, mild cognitive impairment; AD, Alzheimer’s disease.

**Table 4:** Univariate ROC curves analysis.

HS vs AD				AD vs SMC			
Elements	AUC	T-tests	Statistical Power (%)	Elements	AUC	T-tests	Statistical Power (%)
Mn/V	0.94044	4.78E-13	100	Mn/Ni	0.93137	2.08E-08	100
Cu/Mn	0.93456	8.87E-13	95	Cu/Mn	0.91422	4.44E-08	91
Mn/Sr	0.91838	1.18E-11	100	Mn/V	0.91176	3.63E-08	100
Mn/U	0.91691	4.72E-10	100	Ca/Mn	0.90074	1.78E-07	87
Ca/Mn	0.90882	7.58E-11	91	Co/Mn	0.89216	4.34E-07	91
Mn	0.89265	1.63E-10	100	Mn	0.89583	8.33E-08	100
V	0.82978	2.50E-07	94	Hg	0.80515	5.59E-05	91
Hg	0.77206	4.17E-05	88	Zn	0.78064	1.41E-04	96
Se	0.71618	2.35E-04	85	Se	0.77145	2.78E-04	91
Mo	0.70588	0.0018186	93	V	0.73897	0.0017877	84
Zn	0.70588	7.71E-04	71	Fe	0.73652	0.0045145	79
U	0.70515	4.98E-04	87				
HS vs SMC				AD vs MCI			
Elements	AUC	T-tests	Statistical Power (%)	Elements	AUC	T-tests	Statistical Power (%)
Sb/V	0.70781	0.0056067	88	Mn/Ni	0.77059	8.45E-04	94
Cu	0.70417	0.01083	83	Hg	0.74706	0.0035936	83
HS vs MCI				MCI vs SMC			
Elements	AUC	T-tests	Statistical Power (%)	Elements	AUC	T-tests	Statistical Power (%)
Mn/V	0.885	1.30E-09	100	Mn/V	0.85417	3.83E-06	100
Se/V	0.87375	3.74E-09	100	Se/V	0.82083	7.29E-05	100
V/Zn	0.8725	7.31E-09	97	V/Zn	0.81667	2.28E-05	93
Mn/Mo	0.85125	1.92E-06	100	Mn	0.80417	2.12E-04	96
Sb/V	0.84125	6.16E-07	100	Se	0.79271	3.99E-04	99
V	0.80875	7.62E-06	99	Zn	0.7375	0.0060502	90
Mn	0.78	1.86E-05	84	V	0.72917	0.0065436	94
Se	0.7475	0.0013699	98				
Mo	0.7225	0.013149	75				
