# Supplementary material for: Distinctive Pattern of Serum Elements During the Progression of Alzheimer’s Disease
Source: Sci Rep. 2016 Mar 9;6:22769. doi: 10.1038/srep22769 (PMC4783774; doi:10.1038/srep22769)

Distinctive Pattern of Serum Elements During the Progression of Alzheimer's Disease

Giuseppe Paglia[1,2], Oto Miedico[2], Adriana Cristofano[3], Michela Vitale[3], Antonella Angiolillo[3], Antonio Eugenio Chiaravalle[2], Gaetano Corso[4*], Alfonso Di Costanzo[3].

[1] Center for Biomedicine, EURAC Research, Via Galvani 31, 39100 Bolzano, Italy

[2] Istituto Zooprofilattico Sperimentale di Puglia e Basilicata, via Manfredonia 20, 71121, Foggia, Italy

[3] Centre for Research and Training in Medicine for Aging, Department of Medicine and Health Sciences "V Tiberio", University of Molise, Via De Sanctis 1, 86100, Campobasso, Italy

[4] Department of Clinical and Experimental Medicine, University of Foggia, Viale L. Pinto 1, 71122, Foggia, Italy

* Corresponding Author

Prof. Gaetano Corso, Department of Clinical and Experimental Medicine, University of Foggia, Viale L. Pinto 1, 71122, Foggia, Italy.

Tel.: +39 0881 588055; Fax: +39 0881 588037

e-mail: gaetano.corso@unifg.it

Table S1. Loading of principal component analysis (PCA). First and second principal components (PC1 and PC2) of Figure 1.

Table S2. Elements and ratios included in the multivariate ROC model of Figure 6.

Figure S1. Potential biomarkers for discriminating between HS and SMC samples and SMC and MCI samples.

Table S1. Loading of principal component analysis (PCA). First and second principal components (PC1 and PC2) of Figure 1.

ELEMENT	PC1	PC2
Mn	0.077	-0.452
Zn	0.246	-0.382
Se	0.251	-0.353
Hg	-0.006	-0.327
Fe	0.270	-0.200
Tl	-0.073	-0.198
Cr	0.053	-0.137
Co	0.314	-0.126
Ca	0.404	-0.032
Sb	0.093	-0.017
Ni	0.221	-0.001
Pb	0.081	0.014
As	0.228	0.024
Cu	0.325	0.040
Be	0.036	0.054
Al	0.151	0.158
Sr	0.254	0.165
Sn	0.177	0.192
Cd	0.195	0.203
Mo	0.159	0.215
V	0.262	0.225
U	0.234	0.267

Table S2. Elements and ratios included in the multivariate ROC model of Figure 6

Elements	AUC	p value (t-test)
Mn	0.89	4.8E-11
Se	0.72	2.4E-04
Zn	0.71	8.1E-04
Cu	0.64	3.7E-02
Fe	0.62	1.6E-01
Ca	0.55	3.6E-01
Cu/Mn	0.93	1.6E-13
Ca/Mn	0.91	1.8E-11
Fe/Mn	0.88	6.3E-09
Cu/Se	0.85	1.4E-08
Mn/Zn	0.85	5.4E-09
Mn/Se	0.84	6.9E-08
Ca/Se	0.81	6.1E-07
Cu/Zn	0.80	7.5E-06
Ca/Zn	0.78	8.5E-06
Cu/Fe	0.70	3.4E-03
Ca/Fe	0.68	1.4E-02
Ca/Cu	0.64	1.1E-01
Fe/Se	0.63	2.8E-02
Fe/Zn	0.59	9.5E-02
Se/Zn	0.54	4.6E-01

Figure S1. Potential biomarkers for discriminating between HS and SMC samples and SMC and MCI samples.

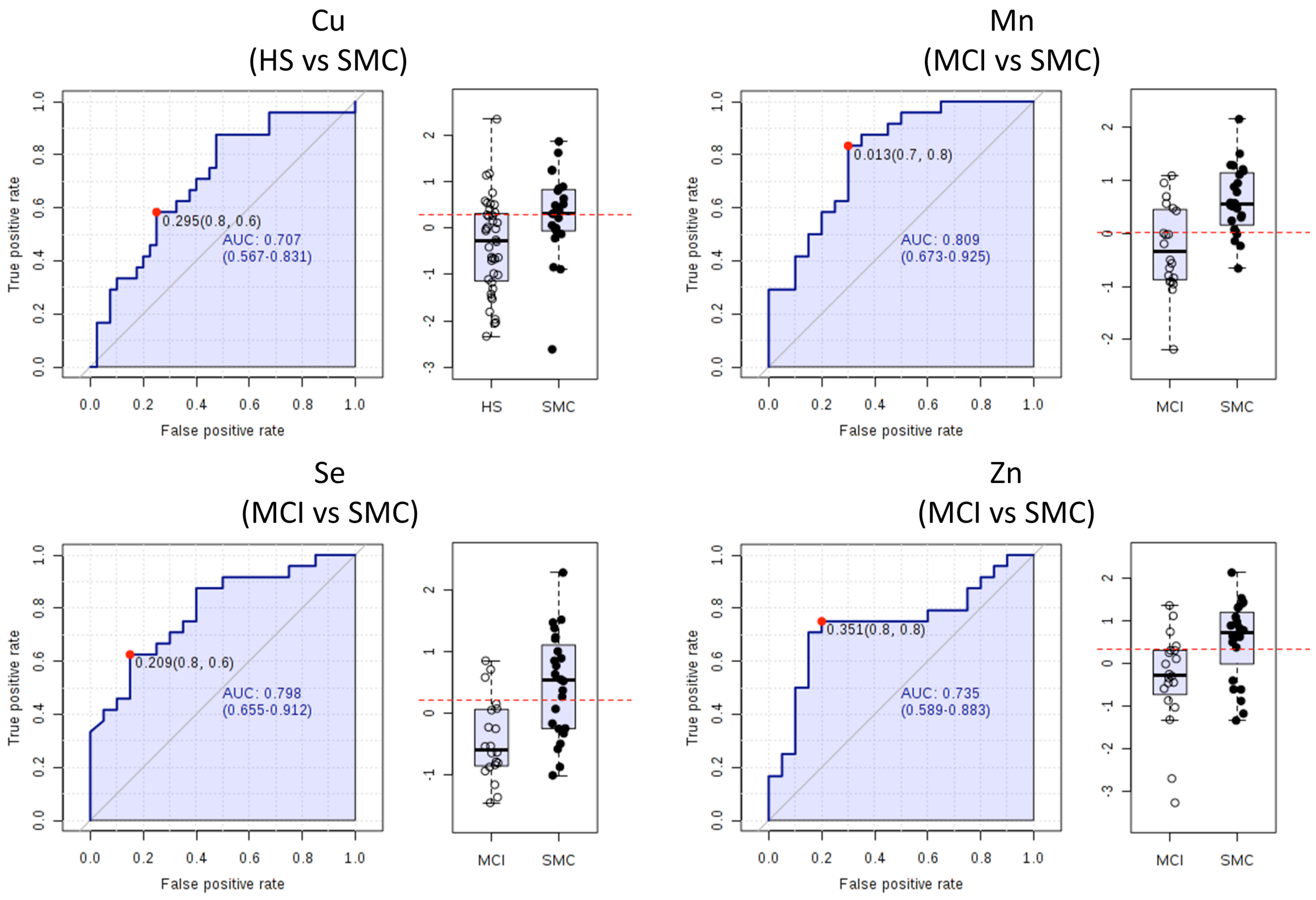

Supplement: Supplementary Information [file srep22769-s1.pdf]
